# Use of the 2D ^1^H-^13^C HSQC NMR Methyl Region to Evaluate the Higher Order Structural Integrity of Biopharmaceuticals

**DOI:** 10.3390/molecules26092714

**Published:** 2021-05-05

**Authors:** Tsang-Lin Hwang, Dipanwita Batabyal, Nicholas Knutson, Mats Wikström

**Affiliations:** Attribute Sciences, Amgen Inc., Thousand Oaks, CA 91320, USA; thwang@amgen.com (T.-L.H.); dbatabya@amgen.com (D.B.); nsk2@g.uclas.edu (N.K.)

**Keywords:** higher-order structure, tertiary structure, fluorescence, circular dichroism, NMR, HOS by NMR, product characterization, biopharmaceuticals

## Abstract

The higher-order structure (HOS) of protein therapeutics is directly related to the function and represents a critical quality attribute. Currently, the HOS of protein therapeutics is characterized by methods with low to medium structural resolution, such as Fourier transform infrared (FTIR), circular dichroism (CD), intrinsic fluorescence spectroscopy (FLD), and differential scanning calorimetry (DSC). High-resolution nuclear magnetic resonance (NMR) methods have now been introduced, representing powerful approaches for HOS characterization (HOS by NMR). NMR is a multi-attribute method with unique abilities to give information on all structural levels of proteins in solution. In this study, we have compared 2D ^1^H-^13^C HSQC NMR with two established biophysical methods, i.e., near-ultraviolet circular dichroism (NUV-CD) and intrinsic fluorescence spectroscopy, for the HOS assessments for the folded and unfolded states of two monoclonal antibodies belonging to the subclasses IgG1 and IgG2. The study shows that the methyl region of the ^1^H-^13^C HSQC NMR spectrum is sensitive to both the secondary and tertiary structure of proteins and therefore represents a powerful tool in assessing the overall higher-order structural integrity of biopharmaceutical molecules.

## 1. Introduction

The higher-order structure (HOS) of proteins includes the secondary, tertiary, and quaternary structure, and represents a critical quality attribute directly related to the structural integrity and the function of therapeutic proteins. The characterization of HOS represents a significant challenge for biopharmaceuticals and is currently being performed using low- to medium-resolution biophysical methods, such as Fourier transform infrared spectroscopy (FTIR), circular dichroism (CD) spectroscopy, intrinsic fluorescence spectroscopy (FLD), and differential scanning calorimetry (DSC) [1,2]. With the increasing interest in different protein modalities in biopharmaceutical development and the rapidly expanding area of biosimilar development, there is a growing need for new analytical methods with higher specificity than the methods commonly applied. During the development and lifecycle of protein therapeutics, the innovator product will most often go through multiple process changes, in which it is required to show that any process-related drug product variations are within the acceptable criteria, and therefore considered comparable. In a similar fashion, it is required to show similarity between the biopharmaceutical reference product and developed biosimilars. The application of nuclear magnetic resonance (NMR) for the assessment of HOS has been suggested as a technology with the potential to more accurately assess differences in HOS as compared to established methods [3]. This technology, referred to as Profile NMR, is based on a one-dimensional diffusion NMR method, in which the strong signals from excipients are efficiently suppressed by dephasing the signals through gradients due to faster Brownian motions of smaller excipient molecules as compared to larger protein in the sample, leaving a spectrum of the protein product only [4,5]. In addition to the 1D NMR method, a 2D ^1^H-^13^C HSQC method was introduced [6], which shows great promise for the HOS assessment of monoclonal antibodies (mAbs) [7]. Finally, mass spectrometric methods, such as hydrogen-deuterium exchange experiments, have also gained considerable interest for the assessment of biopharmaceuticals [8,9].

In this study, we have compared two established methods, near-ultraviolet circular dichroism (NUV CD) and intrinsic fluorescence (FLD) spectroscopy, for the assessment of HOS for biopharmaceuticals against a 2D ^1^H-^13^C HSQC NMR method modified to suppress signals from excipients. To demonstrate the effect HOS has on each spectroscopic method, we compared the folded and unfolded states of two monoclonal antibody subclasses, IgG1 and IgG2, with about 95% sequence identity.

## 2. Results

**Figure 1:**
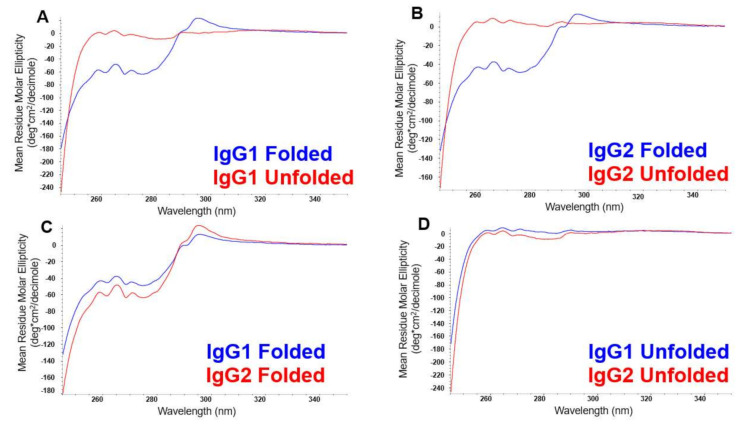
NUV-CD spectra of the folded and unfolded samples of IgG1 (**A**) and IgG2 (**B**). Comparison of the spectra from the folded states of the IgG1 and IgG2 molecules in (**C**), and the unfolded states for these two molecules in (**D**).

The NUV-CD spectra of the folded and unfolded states of IgG1 and IgG2 are shown in Figure 1. The effects of HOS on the differential absorption of left and right circularly polarized light can be seen in the spectral comparisons of the folded and unfolded states of IgG1 and IgG2, in Figure 1A,B, respectively. In general, the NUV-CD spectra of native proteins are characterized by distinct features at around 293 and 286 nm attributable to tryptophan, at 285 to 270 nm attributable to tyrosine and tryptophan, and 250–265 nm attributable to phenylalanine, superimposed over the disulfide signal from 250 to 280 nm. While the unfolded spectra of both IgG’s show relatively featureless lines close to zero (Figure 1D), the folded spectra show absorption changes for the chromophores: tryptophan, tyrosine, and phenylalanine, indicating that these pendent groups are incorporated into highly organized portions of the protein, i.e., tertiary structure. Furthermore, even small differences in HOS and primary structure give rise to unique spectra for the folded states of the two mAbs, allowing them to be distinguished from each other as well (Figure 1C).

**Figure 2:**
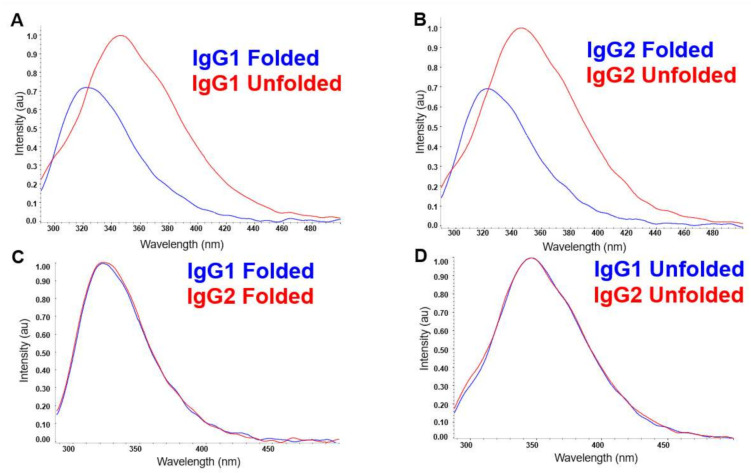
FLD spectra of the folded and unfolded samples of IgG1 (**A**) and IgG2 (**B**). Comparison of the spectra from the folded states of the IgG1 and IgG2 molecules in (**C**), and the unfolded states for these two molecules in (**D**).

The FLD spectra of the folded and unfolded states of IgG1 and IgG2 are shown in Figure 2. The emission wavelengths of the internal fluorophores: tryptophan, phenylalanine, and tyrosine, are sensitive to the polarity of their environments. Higher polarity environments, particularly water from the solvent, cause the wavelengths of emission to lengthen (i.e., red shift). Therefore, unfolded proteins with more solvent-exposed fluorophores will appear more red-shifted than proteins whose tertiary structure tends to sequester these fluorophores in internal, more non-polar environments (Figure 2A,B) [10]. In our study, for both the mAbs IgG1 and IgG2, the folded spectra have a peak around 323 nm, and upon unfolding, the peak shifts to around 345 nm. We also observe that the fluorescence intensity upon unfolding increases (by almost 30%) for both mAbs and is due to the fact that the fluorescence quenching groups are further apart in the unfolded protein than in the native protein, resulting in significant lowering of energy transfer efficiency in the native protein [11]. However, little else about the HOS of the mAbs can be seen by their essentially indistinguishable folded spectra (Figure 2C). In addition, as clearly indicated in Figure 2D, the unfolded spectra for the two mAbs are essentially identical.

**Figure 3:**
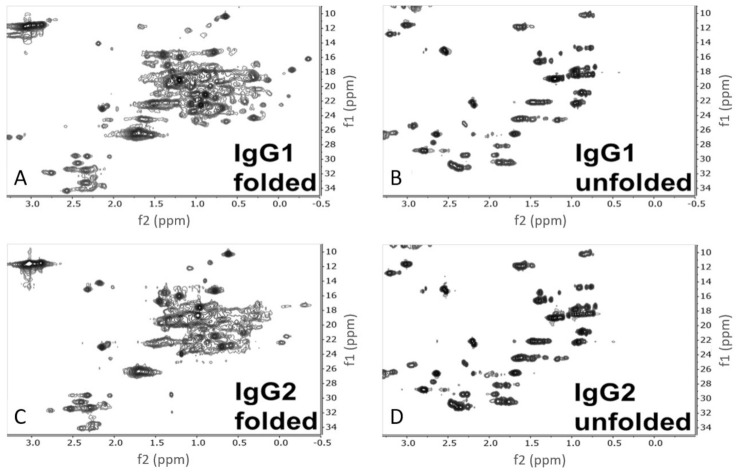
2D ^1^H-^13^C HSQC spectra of the folded and unfolded samples of IgG1 and IgG2. (**A**,**B**) show IgG1 in the folded and unfolded state respectively, whereas (**C**,**D**) show IgG2 in the folded and unfolded states respectively. ^1^H is represented on the x axis (f2), and ^13^C is represented on the y axis (f1).

**Figure 4:**
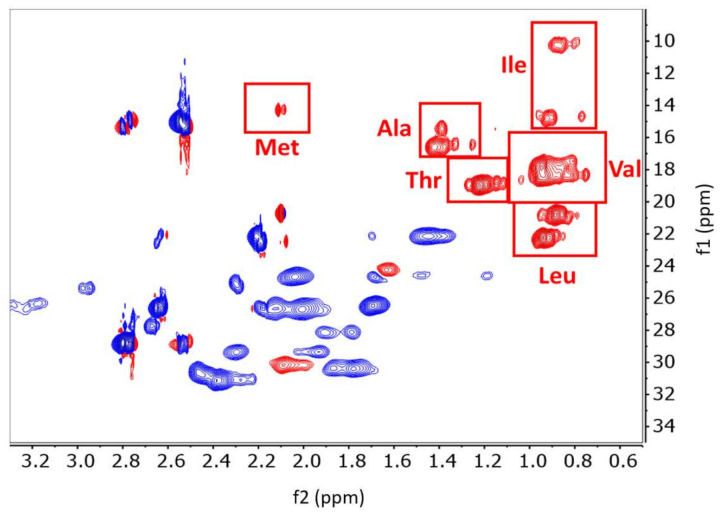
Multiplicity-edited 2D ^1^H-^13^C HSQC spectra of the unfolded state of IgG2. Red peaks are either CH or CH_3_, blue peaks are CH_2_. The methyl groups of each amino acid are shown in red boxes corresponding to the random coil ^1^H and ^13^C shift ranges for the six methyl groups [12]. ^1^H is represented on the x axis (f2), and ^13^C is represented on the y axis (f1).

The ^1^H-^13^C HSQC NMR methyl spectra of the folded and unfolded states of IgG1 and IgG2 are shown in Figure 3. NMR relays information about the local magnetic environments of the nuclei under investigation both through chemical bonds and spatially by the other atoms surrounding them. Atoms in more magnetically shielded environments have lower chemical shifts (plotted in ppms), while atoms in less shielded environments have higher chemical shifts. 2D ^1^H-^13^C HSQC NMR experiments are designed to correlate both protons (*x*-axis) and the carbon-13 atoms (*y*-axis) that they are directly attached to in a molecule. Since proteins are almost entirely composed of protons and carbons, NMR provides a wealth of information about primary structure and all levels of HOS. The resolution of primary structure can be seen in the unfolded spectra of IgG1 and IgG2 (Figure 3B,D). In the absence of any ordered secondary or tertiary structure, the unique chemical signals of different amino acids are resolved and by comparison to reported random-coil (unfolded) chemical shifts, side-chain units, particularly the methyl groups, can be tentatively assigned (Figure 4) [12]. In the folded state, the various magnetic environments of each individual amino acid disperse the side-chain signals to produce a truly unique spectrum for each protein, which is dependent upon all levels of HOS, as shown in Figure 3A,C.

## 3. Discussion

In this study, we have compared the ability of NUV-CD, FLD, and 2D NMR to measure HOS in two monoclonal antibody subclasses, IgG1 and IgG2. Unlike NUV-CD and FLD, which are only able to infer structural integrity from a limited number of chromophores in a protein, 2D NMR provides structural information about the entire molecule and is hence sensitive to even subtle changes in all levels of HOS. If low-resolution spectroscopic methods such as NUV-CD and FLD currently set the bar for assessing the structural integrity of biopharmaceuticals, we propose that vastly more informative 2D ^1^H-^13^C HSQC NMR methods become a replacement in many cases for this type of HOS assessments, and for the product characterization of biopharmaceuticals.

## 4. Materials and Methods

### 4.1. Sample Preparation

The test solutions were prepared from 100 mg/mL monoclonal antibodies IgG1 and IgG2 in the formulation buffer: 10 mM sodium acetate buffer, 9% (*w/v*) sucrose, at pH 5.2. The sequence identity comparing the IgG1 and Ig2 antibodies is 95% [13]. The IgG1 molecule harbors glycosylation on N302, while the IgG2 molecule contains glycosylation modifications on N298. Stock solutions of intact (folded) IgG1 and IgG2 were prepared at 50 mg/mL in the same formulation buffer. Stock solutions of the denatured (unfolded) IgG1 and IgG2 were prepared at 50 mg/mL in the formulation buffer, with 6M urea and 50 mM Tris (2-carboxyethyl) phosphine hydrochloride (TCEP).

### 4.2. Intrinsic Tryptophan Fluorescence Spectroscopy

Intrinsic tryptophan fluorescence spectra were obtained using an Applied Photophysics qCD Chirascan equipped with a fluorimeter at ambient temperature using cuvettes with a path length of 1 cm. Samples were run with an excitation wavelength of 280 nm, an excitation bandwidth of 5 nm, boxcar width of 5 nm, and averaged over 10 scans, with each scan taking 1 s. All mAb samples were diluted to approximately 0.033 mg/mL with buffer before measurements. Each sample was measured in triplicate and buffer blanks were subtracted before data analysis. The spectra were overlaid with each other and the similarity was compared by calculating the variability of the maximum fluorescence intensity and the wavelength at the maximum fluorescence.

### 4.3. Near-Ultraviolet Circular Dichroism Spectroscopy

The NUV-CD spectra were obtained on an Applied Photophysics qCD Chirascan spectropolarimeter at ambient temperature. The protein samples were analyzed at a concentration of about 0.5 mg/mL (both folded and unfolded). Using cuvettes with a pathlength of 1 cm, the spectra were corrected for concentration and contributions from the buffer and are reported as Mean Residue Molar Ellipticity. Each spectrum is an average of 4 scans and was smoothed with a 7-point smoothing function using the OMNIC 32 software (Thermo Fisher Scientific Inc.). Background nitrogen blanks and buffer blanks were measured to eliminate the signals from the nitrogen, cuvette, and buffer. The parameters for the Near UV CD were: 1 cm path length, 240–350 nm wave range, 2 s exposure time, 1 nm bandwidth, 0.5 nm step, and averaged over 4 runs with a 900 μL sample volume.

### 4.4. Nuclear Magnetic Resonance

A Bruker Avance III 600 MHz NMR spectrometer equipped with a 5 mm CPTCI cryoprobe was used to acquire NMR data at 310 K (37 °C) Bruker Biospin Corp, Billerica, MA, USA). Samples were prepared in 5 mm step-down NMR tubes (Wilmad LabGlass, Vineland, NJ, USA) with 5% D_2_O. A modified 2D gradient-selected, sensitivity-enhanced ^1^H-^13^C HSQC NMR method [14] with additional excipient signal suppression was used to acquire the methyl fingerprints of the samples. The WET scheme [15] was used to suppress the acetate signal, and the asymmetric adiabatic pulse (HS1/2, R = 10, 0.9 Tp; tanh/tan, R = 50, 0.1 Tp), with pulse length 375 μs [16], was applied to suppress the carbon signals of the sucrose while exciting the methyl ^13^C signals of the protein. 2D ^1^H-^13^C HSQC experiments for Figure 3 used the following parameters to acquire NMR data: The f_2_ spectral width was 14 ppm centered on 4.7 ppm with 2048 points. The f_1_ spectral width was 28 ppm centered on 21 ppm. Spectra were acquired with 128 increments with 50% non-uniform sampling and 2048 scans in each increment, with recycle delay 0.5 s between scans. The total experimental time was 26.5 h for each spectrum. Digital filtering for 0.4 ppm bandwidth was used to further remove the water signal. GARP decoupling was applied during the WET scheme with 2.08 kHz RF power and the t2 acquisition with 4.16 kHz RF power. Shifted sine-squared bell window functions and zero filling were applied to both dimensions before Fourier transform of the data. The final spectra were 4 k × 1 k. The spectrum in Figure 4 was acquired using the ^1^H-^13^C multiplicity-edited HSQC (hsqcedetgpsisp2.2 in the Bruker library). The f_2_ spectral width was 9 ppm, centered on 4.5 ppm with 2048 points. The f_1_ spectral width was 160 ppm centered on 80 ppm. The 2D data were obtained with 512 increments and 16 scans in each increment, with recycle delay 1 s between scans. The total experimental time was 2.8 h. The data processing was carried out using the spectrometer software (TopSpin, Bruker BioSpin Corp, Billerica, MA, USA) and Mnova software (Mestrelab Research S.L., Santiago de Compostela, Spain).

## 5. Conclusions

The results show that 2D ^1^H-^13^C HSQC NMR is incredibly sensitive to primary, secondary, tertiary, and quaternary structures, and provides unique fingerprints for both the IgG1 and IgG2 subclasses used. Near-ultraviolet circular dichroism (NUV-CD) is also able to differentiate between the two IgG subclasses, while intrinsic fluorescence (FLD) is only able to distinguish between the folded and unfolded states of each protein, but not able to distinguish IgG1 from IgG2. When the 2D NMR methyl fingerprints are visually compared to the results from NUV-CD and FLD, the degree of HOS information captured by 2D NMR is vastly superior to that of either currently established method. Our findings therefore exemplify the superiority of NMR in the assessment of higher-order structural attributes of biopharmaceuticals.

## Abbreviations

HOS	Higher-order structure
HOS by NMR	higher-order structure by nuclear magnetic resonance
FTIR	Fourier transform infrared
NUV-CD	near-ultraviolet circular dichroism
FLD	intrinsic fluorescence spectroscopy
NMR	nuclear magnetic resonance
ppm	parts per million
mAb	monoclonal antibody
